# Potential Health Risk Assessment of Different Heavy Metals in Wheat Products

**DOI:** 10.22037/ijpr.2019.1100865

**Published:** 2019

**Authors:** Kiandokht Ghanati, Farid Zayeri, Hedayat Hosseini

**Affiliations:** a *Food Safety Research Center, Shahid Beheshti University of Medical Sciences, Tehran, Iran. *; b *Department of Food Science and Technology, National Nutrition and Food Technology Research Institute, *; c *Faculty of Nutrition Sciences and Food Technology, Shahid Beheshti University of Medical Sciences, Tehran, Iran. *; d *Department of Biostatistics and Proteomic Research Center, Faculty of Paramedical Sciences, Shahid Beheshti University of Medical Sciences, Tehran, Iran.*

**Keywords:** Health risk assessment, Heavy metal, Wheat products, Inductively coupled plasma-atomic emission spectrometry, Atomic absorption spectrometry

## Abstract

In the present work, health risk of heavy metals such as As, Cd, Co, Cr, Cu, Hg, Ni, Pb, and Zn in Iranian urban and rural samples including wheat, wheat flour, bread, pasta and sweets were assessed. The real amount of heavy metals in target samples were determined by inductively coupled plasma-atomic emission spectrometry (ICP-AES) and atomic absorption spectroscopy (AAS). Wet ashing and hydride generation techniques were used in sample preparation step. Results demonstrated that heavy metal contaminations in cereal samples were significant. The average concentrations of heavy metals in wheat products were between 0.01 mg kg^−1^ to 46 mg kg^−1^. Finally, the health risk assessment results showed that heavy metal contents in rural samples were higher than those in urban samples. The risk of Cu and Zn was significant in two areas and risk of Cr and Cd was not significant.

## Introduction

Heavy metals, such as Lead (Pb), cadmium (cd), iron (Fe), copper (Cu), manganese (Mg), zinc (Zn), and etcrepresent are one of the main sources of pollutant and toxicology in the world (1-3). Some of these heavy metals are essential for human life but excessive amounts of them in the body cause the serious health risks such as cancer and damage to the nervous system (4-7). Excessive presence of Pb in body can affect brain activity in children and surplus of Cd can cause kidney stones (8, 9). Food and Agriculture Organization (FAO) and World Health Organization (WHO) have considered the maximum level for heavy metals in different samples (10).

Cereal and its products such as wheat, wheat-flour, bread, pasta, and sweets are among the biggest groups in food chain. These foods are being used daily and have direct effects on human health (11). In their natural form, cereals are a rich source of vitamins, minerals, carbohydrates, fats, oils, and protein. In many countries, cereal constitutes a majority of daily sustenance. In developed countries, cereal consumption is moderate and varied but still substantial.

There are different ways for heavy metals to enter human body including direct ingestion, dermal contact, diet through the soil-food chain, inhalation, and oral intake (12-16). Food is one of the primary sources for entrance of heavy metals to the body and the knowledge of background values of heavy metals in food such as cereal sample as main nutrition is necessary (17, 18). A cereal sample can get polluted directly from agricultural soil because soil is an important way for heavy metals to be transferred to agricultural products (19). Contamination of heavy metals in soil can be caused in many ways, such as irrigation water, industrial emissions, and the use of manure. Considering the above mentioned facts, health risk assessment of heavy metals and determining an accurate and reliable concentration of them in cereal samples is necessary. The factors affect the presence of heavy metals in cereal samples such as type of cereal samples, type of related soil, area, and etc., should be considered in assessing the procedure. 

Inductively coupled plasma-atomic emission spectrometry (ICP-AES) and flame or graphite furnace atomic absorption spectrometry (F-GFAAS) are fast and highly sensitive instrumentation methods for determining real amounts of heavy metals in various samples (20-23). The other advantages of these methods are good linearity of calibration curves, low detection limits, high recovery, ease of use, and low matrix interferences. 

Prior to instrumentation section, in order to eliminate and decrease the interferences, sample preparation is necessary as the critical step in the analytical process. This stage plays an important role in digestion of complex matrices, cleanup of analyte from co-existing species, analyte extraction and in increasing its sensitivity and recovery. The dry ashing, wet digestion procedures are general methods for this aim. These methods were used to determine many elements in different complex samples (24-26). For determining As and Hg hydride generation is employed as a suitable method for sample preparation step (27, 28).

In this research, we assessed the risk of heavy metals for some elements such as As, Cd, Co, Cr, Cu, Hg, Ni, Pb, and Zn in different urban and rural cereal samples. Golestan, Zarshouran (Kordestan) and Takab (Azarbayjan Gharbi) were selected to obtain cereal samples. Inductively coupled plasma-atomic emission spectrometry (ICP-AES) and atomic absorption spectrometry (AAS) were used as efficient methods for determining heavy metals. The concentration of heavy metals and total heavy metals was reported. Health risk assessment of heavy metals according to type and region (urban and rural) of the cereal was different. 

## Experimental

*Samples Collection*

A total of 28 cereal samples (including 12 wheat, 3 wheat-flour, 2 bread, 6 pasta, and 5 sweets) were collected from Golestan, Zarshouran (Kordestan) and Takab (Azarbayjan Gharbi) in June, 2016. All the samples were grinned, kept in appropriate vessel and transported to the laboratory. A representative sample of each cereal was properly stored in closed bottles in ambient temperature to determine heavy metal contents.

*Reagents*

As, Cd, Co, Cr, Cu, Hg, Ni, Pb, and Zn were purchased from Sigma-Aldrich (Steinheim, Germany) at a purity higher than 99%. The mixed standard solution was made at concentration of 100 μg kg^−1^. Working solutions were prepared by diluting stock solution with double deionized water (Milli-Q Millipore 18.2 MΩ/cm resistivity) for linear range assay. HNO_3_, H_2_SO_4_, H_2_O_2_, HF, HClO_4_, and HCl were obtained from Merck Co. (Darmstadt, Germany). All the plastic and glassware were cleaned by soaking in dilute HNO_3_ and were rinsed with distilled water prior to use. 

*Digestion Procedure*

The samples were prepared according to AOAC 986.15 and AOAC 999-11. They were digested as follows: 

Analysis of As and Hg (Hydride generation procedure): A 0.3 g sample was added to a 50 mL round-bottom flask. Then, 5 mL concentrated nitric acid was mixed to the sample and heated for 60 min in 150 °C. Then, 1 mL magnesium nitrate at a concentration 75 mg L^-1^ was added and heated in 450 °C for dehydration. After this step, 2 mL HCl (8 moL L^-1^) was added and thoroughly shacked. Finally, 200 µL potassium iodide (1% w/v) was added and this sample solution was diluted up to a 25 mL in the volumetric flask with distilled water. The obtained sample solution was immediately introduced to the instrument. 

Analysis of the other elements: 5 g sample was weighted and 20 mL HNO_3_ 10% was added and shook. Then, this sample was heated in 100 °C for 2 h for thorough dehydration. This sample was overheated in 350 °C by heater and then introduced to furnace for 5-6 h in 200 °C. After cooling, 2 mL concentrated HNO_3_ was added and heated to the complete dehydration. This sample was placed in furnace again for 2 h in 450 C to get white ash. Then, 5 mL HCl 6 moL L^-1^ was added and heated for dehydration. Finally, 10 mL concentrated HNO_3_ was added and introduced to the instrument.

*Instrumentation*

Thermo Scientific iCAP Series 6500, equipped with a charge injection device (CID) detector CETAC and Asx-520 Autosampler (England) has been used for determination of the elements. Control of the spectrometer is provided by PC based iTEVA software. The metals were determined with inductively coupled plasma-atomic emission spectrometry (ICP-AES). A model 220Z graphite furnace atomic absorption spectrometer equipped with Zeeman background (Varian, Australia) and pyrolytic partitioned graphite tubes (Varian, Australia) were used. Argon was used as inert gas at the flow rate of 3.0 L min^-1^ in all stages except for the step of atomizing of which flow was stopped. In this study, for the cases in which simultaneous ICP-OES, and in those samples in which ICP-AES was insufficiently sensitive, concentrations of the metals were determined by a GF-AAS. The determinations of Hg and As were performed using a Varian SpectrAA 220 atomic absorption spectrometer (Varian, Australia) equipped with a Varian GTA-110 graphite furnace and hydride generation-atomic fluorescence. Pyrolytic-coated graphite tubes with a platform were used and the signals were measured as peak areas. 

*Statistical Analysis*

The data were analyzed by independent student’s t-test with SPSS version 15.0 for windows and the differences were considered statistically significant at *P* < 0.05.

*Health Risk Analysis*

The human risk (non-cancer) effects for all the metals were assessed. Equation 1 shows the Chronic Daily Intake (CDI) (mg kg^−1^ day^−1^). In this equation CF is the median concentration of HM in the sample (mg kg^−1^), IR is the ingestion rate of the sample (kg person^−1^ day^−1^), EF is exposure frequency (365 days year^−1^), and ED is the exposure duration. CDI is related to CF, IR, EF, and BW, but usually body weight (BW: 61.6 kg for adults) and exposure duration (ED: 365 days years^-1^) is constant in each region and CF and IR have the most role. 

$\mathrm{DI}\left({\mathrm{mgkg}}^{-1}{\mathrm{day}}^{-1}\right)=\frac{\mathrm{CF}\times \mathrm{IR}\times \mathrm{EF}\times \mathrm{ED}}{\mathrm{BW}\times \mathrm{AT}}$

Equ. 1

Risk to human health by the intake of metal-contaminated food was characterized using a hazard quotient (HQ) (US EPA, 1989). HQ is the ratio between exposure and the reference oral dose (RfD). If CDI increases the HQ increases (equation 2) and if HQ goes above one, there is cause for concern. If the ratio is lower than one (1), then there is no apparent risk. An estimate of the potential hazard to human health (HQ) through consumption of wheat grain grown in metal-contaminated is described in Equation 2.

Equ. 2 $$\mathrm{HQ}=\frac{\mathrm{CDI}}{R_{f}\mathrm{DO}}$$

To evaluate the potential risk to human health through more than one HM, the hazard index (HI) has been developed (US, 1986). The hazard index, that is the sum of the hazard quotients assumes that the magnitude of the adverse effect will be proportional to the sum of multiple metal exposures. It also assumes similar working mechanisms that linearly affect the target organ. When the hazard index exceeds 1.0, there should be concern for potential health effects.

$\mathrm{HI}=\sum_{n=1}^{i}{\mathrm{HQ}}_{n}$

## Results

In this work, health risk of heavy metals in Iranian urban and rural area (Golestan, Zarshouran (Kordestan, and Takab (Azarbayjan Gharbi)) were assessed in the samples including wheat, wheat flour, bread, pasta, and sweets in order to evaluate health risks hazards of non-cancerous diseases through exposure to the selected samples by the local inhabitants, CDI, HQ and HI, were determined according to EPA’s Guidelines for Health Risk Assessment of Chemical Mixtures (US 1986). 

Table 1 shows the results. Except for CDI for Co in rural sample, CDI was lower than RfDO for whole target elements in urban and rural samples.

**Figure 1:**
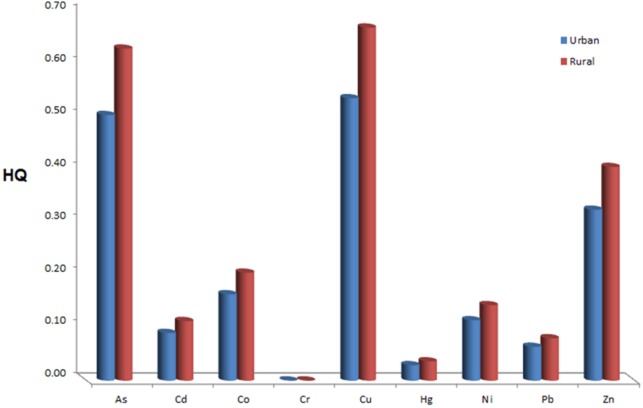
The comparison of hazard quotient (HQ) between urban and rural sample

Figure 1. shows the comparison between heavy metals HQ for rural and urban sample in this order: Cu>As>Zn>Co>Ni>Cd>Pb>Hg>Cr. HQ for rural population is higher than urban population. HI 1.83 was for urban and HI 2.28 was for rural samples. According to the results, the rural samples had higher HI than the urban samples and HI was significant for two areas.

*Levels of heavy metals in cereal samples*

Table 2 shows the concentration of heavy metals in wheat samples. Zn and Cu have the maximum concentrations, respectively, and the concentration of Zn is quite significant. The average of heavy metals concentrations in wheat increased in the order Cd>Co=Hg >Pb>Cr>As>Ni>Cu>Zn>. The concentration of heavy metals in wheat was between >0.001 to 36.3 mg kg^-1^.

Table 3 describes the concentration of heavy metals in wheat-flour, pasta, bread, and sweets. According to these results, the amounts of heavy metal especially Zn, were significant in these samples. The average concentration of heavy metals for wheat-flour was > 0.01-19.9, pasta: 0.019-29.6, bread: >0.01-25.8, and sweets: 0.018-13.9. In all of samples the concentration of Zn and Cu was significant.

## Discussion

According to Table 1, CDI was lower than RfDO for whole target elements (except Co) in urban and rural samples. CID for rural sample is higher than urban sample but this difference is not significant. The highest and lowest HQ for rural region was directly related to Cr and for urban area was related to Cu. There is significant difference between type of heavy metals for HQ with maximum 0.67 and minimum 0.0001 for Cu in the rural sample and Cr in the urban sample. But there is no significant difference for single element in the urban and rural areas. In any case, the value of hazard quotient (HQ) of any element is less than one, which means that there are no carcinogenic threats for any individual element. The potential risk of heavy metals could be increased when they were considered together at the same time but this risk was not significant when each metal was individually analyzed. HI values were 2.28 and 1.83 for rural and urban groups, respectively. The health risk of heavy metals for rural was higher than urban. It can be related to the more sources of entire heavy metals in the rural areas than in the urban areas. Therefore, the health of rural and urban residents through wheat consumption should be considered as a potential threat from heavy metals in Iran. 

Table 2 and Table 3 show the mean concentrations of heavy metal in wheat, wheat-flour, bread, pasta, and sweets samples. The results show that the concentration of Zn and Cu in five groups of the samples was significant. For wheat samples, heavy metal with the lowest concentration was Cd and Co. The highest level of Zn and lowest level of Cd may be associated with physicochemical characterization of soil from which wheat was harvested. Ni and Hg have the lowest concentration in wheat-flour/bread and pasta/sweets, respectively. The results demonstrate that the amount of each heavy metal in five groups of the samples was variable in a wide range and this was affected from different factors such as type of heavy metals, physicochemical properties of related soil, region, type of products, etc,.

**Table 1:** Chronic daily intake (CDI), oral reference dose (RfDO), hazard quotient (HQ) and total exposure hazard index (HI) for urban and rural samples

**Element**	**Area**	**CDI**	**RfDO**	**HQ**	**HI**
	Urban	0.0002	0.0003	0.50	1.83
As					
	Rural	0.0002		0.63	2.28
	Urban	0.0001	0.001	0.09	1.83
Cd					
	Rural	0.0001		0.11	2.28
	Urban	0.0000	0.0003	0.16	1.83
Co					
	Rural	0.0001		0.20	2.28
	Urban	0.0005	1.5	0.0001	1.83
Cr					
	Rural	0.0006		0.00	2.28
	Urban	0.0214	0.04	0.54	1.83
Cu					
	Rural	0.0268		0.67	2.28
	Urban	0.0001	0.0003	0.03	1.83
Hg					
	Rural	0.0001		0.04	2.28
	Urban	0.0023	0.02	0.11	1.83
Ni					
	Rural	0.0029		0.14	2.28
	Urban	0.0079	0.4	0.06	1.83
Pb					
	Rural	0.0098		0.08	2.28
	Urban	0.0973	0.3	0.32	1.83
Zn					
	Rural	0.1216		0.41	2.28

**Table 2:** Summary of heavy metal concentrations (mg kg−1) in the wheat samples

**Sample**	**Code**	**As**	**Cr**	**Co**	**Ni**	**Cu**	**Zn**	**Cd**	**Hg**	**Pb**
Wheat	1	> 0.05	0.175	0.015	0.234	3.380	21.142	0.012	0.018	>0.01
Wheat	2	0.295	0.130	0.0225	0.288	3.263	36.363	0.036	0.020	0.053
Wheat	3	0.131	0.177	0.016	0.298	2.853	22.750	0.014	0.026	0.289
Wheat	4	0.142	0.075	0.022	0.324	3.107	27.941	0.024	0.022	0.073
Wheat	5	0.111	0.069	0.017	0.290	2.557	16.964	0.014	0.026	>0.01
Wheat	6	> 0.05	0.065	0.013	0.327	2.553	16.888	0.015	0.020	0.007
Wheat	7	0.227	0.081	0.022	0.256	3.858	29.973	0.028	0.024	0.061
Wheat	8	> 0.05	0.072	>0.01	>0.01	0.016	11.787	0.002	0	>0.01
Wheat	9	0.114	0.042	0.015	0.309	2.320	18.120	0.010	0.010	>0.01
Wheat	10	0.092	0.053	0.014	0.358	2.926	23.19	0.017	0.013	>0.01
Wheat	11	0.071	0.100	0.031	0.337	2.208	11.695	0.009	0.011	>0.01
Average		0.111	0.094	0.017	0.275	2.640	21.528	0.016	0.017	0.044
Recovery(%)		85	94	89	87	92	99	96	88	89
RSD(%)		4.3	5.7	6.8	5.2	4.4	7.0	8.6	5.5	6.1

**Table 3:** Summary of heavy metal concentrations (mg kg^−^^1^) in the wheat-flour, pasta and sweets samples

**Pb**	**Hg**	**Cd**	**As**	**Zn**	**Cu**	**Ni**	**Co**	**Cr**	**Sample code**
0.063	0.009	0.477	6.170	23.133	0.036	>0.01	0.063	0.152	Wheat-flour 1
0.083	0.029	0.821	12.280	19.902	0.026	>0.01	0.079	0.27	Wheat flour 2
0.049	0.025	0.558	7.202	16.678	0.027	>0.01	0.060	0.164	Wheat-flour 3
0.065	0.021	0.619	8.551	19.904	0.030	>0.01	0.068	0.198	Average
90	89	91	82	89	94	90	85	88	Recovery (%)
4.7	5.7	7.1	4.9	5.0	6.8	8.6	5.3	6.0	RSD (%)
0.250	0.015	0.373	3.404	12.974	0.091	0.021	0.051	1.130	Pasta 1
0.289	0.018	0.401	4.935	13.960	0.058	0.024	0.034	0.694	Pasta 2
0.175	0.019	0.328	6.453	42.597	0.670	0.033	0.030	0.252	Pasta 3
0.188	0.019	0.338	5.935	27.328	0.075	0.014	0.023	0.228	Pasta 4
0.164	0.022	0.352	6.070	46.764	0.809	0.038	0.029	0.260	Pasta 5
0.136	0.018	0.300	5.780	34.037	0.242	0.017	0.022	0.071	Pasta 6
0.200	0.019	0.349	5.429	29.610	0.324	0.025	0.032	0.439	Average
99	81	88	96	97	82	89	94	90	Recovery (%)
5.9	6.9	6.8	7.6	8.2	5.3	6.6	6.5	7.1	RSD (%)
0.109	0.026	0.601	6.548	17.505	0.028	>0.01	0.062	0.405	Bread 1
0.059	0.023	0.530	10.323	34.237	0.039	>0.01	0.075	0.303	Bread 2
0.084	0.024	0.565	8.436	25.871	0.033	>0.01	0.068	0.354	Average
93	88	91	89	95	90	84	86	91	Recovery (%)
6.2	4.4	5.6	4.7	6.1	5.9	4.9	5.0	5.1	RSD (%)
0.063	0.009	0.477	6.170	23.133	0.036	>0.01	0.063	0.152	Sweets 1
0.083	0.029	0.821	12.280	19.902	0.026	>0.01	0.079	0.27	Sweets 2
0.049	0.025	0.558	7.202	16.678	0.027	>0.01	0.060	0.164	Sweets 3
0.065	0.021	0.619	8.551	19.904	0.030	>0.01	0.068	0.198	Sweets 4
0.250	0.015	0.373	3.404	12.974	0.091	0.021	0.051	1.130	Sweets 5
0.289	0.018	0.401	4.935	13.960	0.058	0.024	0.034	0.694	Average
84	99	86	93	82	88	96	95	87	Recovery (%)
6.3	4.8	6.0	5.8	5.5	5.7	6.3	4.9	5.0	RSD (%)

## Conclusion

In this study, we successfully determined the amount of heavy metals in different wheat samples using high sensitive inductively coupled plasma-atomic emission spectrometry (ICP-AES) and atomic absorption spectrometry (AAS). Digestion method as effective sample preparation was employed for decreasing the interference of the sample matrix and decreasing the sensitivity. Also, this research provides a comprehensive assessment of heavy metal pollution from urban and rural sample in Iran. The results demonstrated that the risk index for target element in urban and rural areas was significant and different. 
